# Stress and fatigue in nurses in critical care units and their association with salivary cortisol

**DOI:** 10.1590/0034-7167-2024-0282

**Published:** 2025-06-20

**Authors:** Dnieber Chagas de Assis, Deisy Vivian de Resende, Robinson Sabino-Silva, Maria Helena Palucci Marziale

**Affiliations:** I Universidade Federal de Uberlândia. Uberlândia, Minas Gerais, Brazil; II Universidade de São Paulo. Ribeirão Preto, São Paulo, Brazil

**Keywords:** Hydrocortisone, Occupational Health, Stress, Physiological, Fatigue, Nurses., Hidrocortisona, Salud Laboral, Estrés Fisiológico, Fatiga, Enfermeras y Enfermeros.

## Abstract

**Objectives:**

to evaluate the association between stress, fatigue and salivary cortisol levels in nurses working in emergency units (EU) and surgical units (SU).

**Methods:**

cross-sectional study conducted in two university hospitals with participation of 66 nurses. The Nursing Stress Inventory and the Fatigue Assessment Scale were applied, and saliva samples were obtained to determine cortisol concentration.

**Results:**

high levels of stress and fatigue were observed in 50.8% and 46% of the professionals, respectively. Nurses in EU had higher levels of stress and fatigue and lower secretion of salivary cortisol during the work shift compared to nurses in the SU, without statistical significance. Cortisol secretion in the morning period was correlated with fatigue (ρ=0.25 and p=0.04).

**Conclusions:**

the results suggest a dysregulation of the hypothalamic-pituitary-adrenal axis in nurses from the EU and a significant correlation between fatigue and cortisol secretion in the morning period.

## RESUMEN

**Objetivos:**

evaluar la asociación entre estrés, fatiga y niveles de cortisol salival en enfermeros de unidades de emergencia (UE) y quirúrgica.

**Métodos:**

estudio transversal en dos hospitales universitarios con 66 enfermeros. Se aplicaron el Inventario de Estrés en Enfermeros y la Escala de Evaluación de la Fatiga, y se obtuvieron muestras de saliva para determinar la concentración de cortisol.

**Resultados:**

se observaron niveles elevados de estrés (50,8%) y fatiga (46%) en los profesionales. Los enfermeros de las UE presentaron niveles más elevados de estrés y fatiga y menor secreción de cortisol salival durante el turno de trabajo en comparación con los enfermeros de la unidad quirúrgica, sin significancia estadística. La secreción de cortisol por la mañana se correlacionó con la fatiga (ρ=0,25 y p=0,04).

**Conclusiones:**

los resultados sugieren una desregulación del eje hipotálamo-hipofisario-adrenal en enfermeros de la UE y una correlación significativa entre la fatiga y la secreción de cortisol por la mañana.

## INTRODUCTION

Cortisol is the main glucocorticoid hormone produced by the adrenal glands after activation of the hypothalamic-pituitary-adrenal (HPA) axis. Its secretion follows a deep circadian rhythm with high levels around thirty minutes after waking up and a gradual decrease throughout the day^(1)^. This hormone plays a central role in several biological processes, including energy metabolism, blood pressure maintenance, immunomodulation, regulation of cognitive and memory functions, and stress response. Clinical and experimental evidence suggests that cortisol concentration may be related to exposure to stress, fatigue, and the development of metabolic diseases, and its measurement in saliva samples has been widely used in occupational stress research^(2-4)^.

Given their long working hours in different shifts, nursing professionals are especially susceptible to chronic stress and fatigue, the consequences of which include physical and mental illness, decreased work capacity, increased occupational accidents, and absenteeism^(5,6)^. Chronic fatigue, drowsiness, and insomnia caused by sleep deprivation/interruption are highly prevalent symptoms in these professionals, directly related to the work shift, and occur more frequently in night shift workers^(7,8)^.

Due to their work characteristics, some hospital sectors have a greater potential for stress risk. Studies have shown that nurses in emergency units (EU) and surgical units (SU) are exposed to additional stressors such as noise, light, radiation, violence, performance of cardiopulmonary resuscitation procedures, and death. Stimuli such as the need for rapid and immediate action in highly complex procedures, overcrowding, and unpredictability make nurses in critical units more susceptible to burnout syndrome, psychosomatic stress, and fatigue than those in other hospital sectors^(3,9,10)^.

Even though the literature presents a consensus on the promotion of misalignment of circadian rhythms and endocrine alterations by occupational stressors, their association with salivary cortisol secretion and the possible impact on health have not yet been fully elucidated^(11-13)^, especially in hospital nurses. The great variability of methodological designs may be a plausible explanation for the inconsistency of the results. The proliferation of cortisol indices, often redundant, and the irrational use of these constructs have prevented comparison between studies^(14,15)^. Additionally, the HPA axis has broad functionality and biological complexity, and is able to regulate fight-or-flight situations and promote long-term behavioral adaptations^(16)^.

In this context, the present study was developed with the aim to expand scientific knowledge about the possible associations between occupational stress, fatigue and salivary cortisol secretion in nurses working in critical areas. It is expected that the results obtained will provide strategies for detecting stress and fatigue in nurses, contributing to the development of measures aimed at improving the physical and mental health of these professionals.

## OBJECTIVES

To evaluate the association between occupational stress, fatigue and salivary cortisol levels in nurses working in the EU and SU.

## METHODS

### Ethical aspects

Based on Resolution number 466 of 2012^(17)^, this study was approved by the Research Ethics Committees of the Escola de Enfermagem de Ribeirão Preto at the *Universidade de São Paulo*, of the *Hospital das Clínicas da Faculdade de Medicina de Ribeirão Preto* at the *Universidade de São Paulo* and the *Universidade Federal de Uberlândia*. Written informed consent was obtained from all individuals involved in the study.

### Study design, period and location

This cross-sectional, quantitative study was conducted in the EU and SU of two public teaching hospitals; one in the state of São Paulo (HSP) and the other in Minas Gerais, Brazil (HMG), between January and March 2017. The recommendations of the Strengthening the Reporting of Observational Studies in Epidemiology (STROBE)^(18)^ were used to guide study design.

### Population; inclusion and exclusion criteria

The population of this study consisted of 191 nurses and non-probabilistic convenience was the sampling method used. Professionals of both sexes who worked morning, afternoon and night shifts in fixed or rotating shift schedules were included. Rotating shifts were considered as working schedules that changed over time, that is, nurses worked their work schedule in both morning and afternoon or night shifts. Nurses who used insulin, antiarrhythmics, psychiatric or weight loss medications, or other medications that could interfere with the sleep/wake cycle or cortisol levels, and individuals diagnosed with periodontal, cardiovascular, liver, psychiatric diseases, and thyroid disorders were excluded^(19-21)^. After applying the inclusion and exclusion criteria, 66 nurses participated in the study.

The first stage of data collection consisted of reading and applying an instrument composed of closed questions regarding sociodemographic characteristics, work activity, health conditions, medication use, lifestyle habits and menstrual cycle. This instrument was created and validated by the authors using the Delphi technique^(22)^, following Spínola’s^(23)^ recommendation of a minimum of 70% inter-rater agreement.

### Study protocol

The second stage of data collection consisted of obtaining saliva samples and applying the questionnaires and both were performed by a member of the research team. The instruments were applied in a private room during each participant’s workday, in the morning, afternoon or night shifts.

The Nursing Stress Inventory (NSI), developed and validated for Brazilian nurses by Stacciarini and Tróccoli^(24)^, and the Fatigue Assessment Scale (FAS), created by Michielsen, de Vries, van Heck, van de Vijver, and Sijtsma^(25)^ in the Dutch context, validated for Portuguese by Gouveia et al.^(26)^ were applied for the assessment of stress. The analysis of the NSI and FSA scores were performed by dividing the overall score by the median, and the stress levels were dichotomized as high (greater than the median) and low (less than or equal to the median), and fatigue as “presence” or “absence”, also based on the median of data. The NSI and the FAS presented Cronbach’s alpha of 0.75 and 0.70, respectively.

Saliva samples were collected in Salivette^®^ tubes (Salimetrics, State College, PA, USA), and instructions to be followed were: do not drink alcohol or perform dental procedures 24 hours before collection; do not brush or floss teeth three hours before collection; do not eat, drink (except water) or smoke 30 minutes before collection. Immediately before collection, check for oral lesions with active or potential bleeding and clean the mouth with water by gently rinsing. Open the Salivette^®^, remove the swab, keep it in the mouth for two minutes and return it to the initial position in the Salivette^®^, closing it firmly. Participants were instructed to keep saliva samples in a refrigerator and, at the time of delivery, transport them in styrofoam containers with dry ice provided by the research team.

Four samples were collected on a single workday: upon awakening (sample 1), 30 to 45 minutes after awakening (sample 2), at the beginning of the work shift (sample 3), and at the end of the work shift (sample 4). Samples 1 and 2 were collected by participants themselves, while the others were collected by a member of the research team. The criterion established by Weitzman et al.^(27)^ of a minimum Cortisol Awakening Response (CAR) of 2.5 nmol/L was adopted. The saliva samples were centrifuged at 1,000×g for two minutes at 4°C to obtain the supernatant, aliquoted, and kept at -70°C until analysis.

Quantitative determination of salivary cortisol was performed using the competitive immunoassay (ELISA) method with the commercial Cortisol Saliva ELISA kit (Diagnostic Biochem Canada Inc, Ontario, Canada) according to the manufacturer’s instructions. Validation of the analytical runs was performed by analyzing the mean and standard deviation of each of the controls (high and low) provided in the kit. The absorbance values of the samples were compared with the standard curve plotted by analyzing the absorbances of the calibrators, and transformed into nmol/L.

The analysis of cortisol secretory activity was performed according to indexes previously described in the literature^(28,29)^. The AUCg index represents the calculation of the area under the curve in relation to zero or ground, and the AUCi index represents the area under the curve in relation to the increase. Cortisol secretion during the work shift (AUCwork) was calculated based on the total area under the curve corresponding to the salivary cortisol concentrations at the beginning and end of each work shift. Total cortisol secretion throughout the workday (AUCCD) was calculated based on the total area under the curve corresponding to all cortisol concentrations throughout the day. Time was computed in decimal hours to calculate the areas.

### Analysis of results and statistics

The collected data were stored in a database created in Microsoft Office Excel version 2010. They were later entered into the Statistical Package for the Social Sciences (SPSS) version 24 for descriptive and inferential analyzes. Numerical variables were categorized to enable statistical analyzes. Mean cortisol levels were compared using the Mann-Whitney test for dichotomous variables and the Kruskal-Wallis test for categorical variables with more than two levels, since the data related to cortisol did not present a normal distribution according to the Shapiro-Wilk test. The Spearman correlation coefficient was used to analyze the correlation between variables. Possible associations were assessed using the Chi-square and Fisher’s exact tests. The significance level adopted was p ≤ 0.05.

## RESULTS

A total of 66 nurses were included in the study, 51.5% (34/66) from the HSP and 48.5% (32/66) from the HMG. Most professionals were female (75.7%; n=50), married (60.6%; n=40), mean age of 38.2 ±7 years. Regarding the work sector, 71.2% (n=47) of nurses worked in the EU and 28.7% in the SU, with a mean of 12.7±7.4 years of experience in the profession and 6.5 ± 5.7 years in the work sector. Regarding work shift, 39.3% (n=26) of the nurses were on the morning shift, 25.7% (n=17) on the afternoon shift and 35% (n=23) on the night shift. Most professionals worked in rotating shifts (66.6%; n=44). Some of the nurses (25.7% (n=17) worked a weekly workload of more than 44 hours.

Regarding health habits, 93.9% (62/66) of the nurses were non-smokers and 56% (37/66) reported practicing some physical activity. Coffee was the most consumed beverage among the participants (81.8%), and of these, 66.6% (36/54) consumed one to four cups per day.

Of the 66 nurses who collected saliva samples, two (3%) exhibited a CAR lower than 2.5nmol/L and were considered non-adherent to the collection protocol. One participant requested to leave the study and his samples were also excluded from the analyzes. Thus, the characterization of stress and fatigue was performed in 95.4% (n=63) of participants, and a significant and positive correlation was found between these variables (ρ=0.36; p=0.03). Approximately half of professionals (50.8%) presented high levels of stress, and interpersonal relationships presented the highest average in the sum of the scores (47.8±10.52). Fatigue, assessed by the FAS, was observed in 46% of nurses.

A high level of stress and fatigue was frequently observed in nurses in emergency units, in professionals working rotating shifts and on morning shifts, without statistical significance (Table 1). Regarding health-related variables, coffee consumption was the only one significantly associated with fatigue in nurses (p=0.023).

**Table 1:** Association between the stress and fatigue variables in relation to the occupational characteristics and health-related habits of nurses, Ribeirão Preto, São Paulo, Brazil, 2023 (N=63)

	Stress			Fatigue		
	**High**	**Low**	***p***	**Presence**	**Absence**	***p***
Workplace						
Emergency Unit	23	22	0.926^1^	20	25	0.783^1^
Surgical Room	9	9		9	9	
Work Schedule						
Fixed	10	11	0.859^1^	7	14	0.187^1^
Rotating	21	21		22	20	
Work Shift						
Morning	11	14	0.743^2^	11	14	0.930^2^
Afternoon	9	7		8	8	
Night	11	11		10	12	
Coffee Consumption						
Yes	24	27	0.337^1^	27	24	0.023^1^
No	8	4		2	10	

1 Chi-square test,

2 Fisher’s exact test.

**Figure 1:**
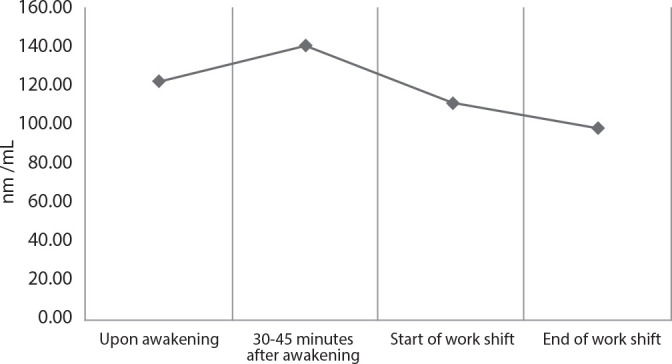
Mean salivary cortisol concentration throughout the workday, Ribeirão Preto, São Paulo, Brazil, 2023

Participants had higher salivary cortisol levels in the morning and a gradual decline throughout the day^(30)^. The mean cortisol concentrations were 122.34 ± 28.19 nmol/L upon awakening; 140.7 ± 27 nmol/L at 30 to 45 minutes after awakening; 110.98 ± 35.04 nmol/L at the beginning of the work shift and 98.03 ± 29.77 nmol/L at the end of the work shift. The mean increase in cortisol upon awakening was 15% (Figure 1).

No statistically significant associations were found between salivary cortisol levels and sociodemographic variables. However, male subjects showed greater HPA axis reactivity upon awakening (AUCi) and greater cortisol secretions during the work shift (AUCwork) and throughout the workday (AUCCD) compared to female subjects (Table 2). Similarly, no statistically significant associations were observed between stress, fatigue, and cortisol levels. However, the AUCg index, which determines total cortisol secretion in the morning, correlated significantly and positively with fatigue (ρ=0.25 and p=0.04).

**Table 2:** Association between salivary cortisol levels, sociodemographic and occupational characteristics of nurses, Ribeirão Preto, São Paulo, Brazil (N=63)

	AUCi	*p*	AUCg	*p*	AUCTrab	*p*	AUCCD	*p*
Sex								
Female	4.79 (±3.70)	0.170^1^	75.36 (±24.73)	0.583^1^	812.63 (±424.76)	0.232^1^	2046.17 (±1242.48)	0.821^1^
Male	6.14 (±4.23)		65.80 (±22.90)		952.66 (±426.30)		2364.02 (±1726.74)	
Stress								
Low	5.54 (±4.08)	0.329^1^	72.53 (±15.00)	0.705^1^	832.76 (±385.73)	0.967^1^	2064.45 (±1324.23)	0.773^1^
High	4.7 (±3.63)		73.62 (±31.07)		858.78 (±467.45)		2177.46 ( ±1421.57)	
Fatigue								
Presence	5.15 (±4.08)	0.983^1^	75.11 (±17.65)	0.247^1^	912.50 (±487.37)	0.432^1^	1998.76 (±1456.25)	0.424^1^
Absence	5.09 (±3.88)		71.36 (±29.01)		789.24 (363.48)		2226.84 (±1294.07)	
Work Schedule								
Fixed	5.92 (±4.43)	0.351^1^	68.91 (±21.68)	0.281^1^	851.71 (±421.92)	0.942^1^	2199.18 (±1457.68)	0.816^1^
Rotating	4.71 (±3.50)		75.16 (±25.54)		843.10 (±432.99)		2083.18 (1332.24)	
Works Shift								
Morning	4.59 (±3.01)	0.763^2^	71.34 (±14.58)	0.522^2^	833.47 (±436.57)	0.981^2^	1896.25 (±1419.61)	0.125^2^
Afternoon	5.04 (±4.24)		78.29 (±24.40)		868.61 (±461.99)		2356.32 (±890.76)	
Night	5.76 (±4.43)		71.27 (32.43)		843.71 (±405.91)		2207.68 (±1586.72)	

1 Mann-Whitney test,

2 Kruskall-Wallis test.

Nurses in the EU had lower levels of salivary cortisol throughout the workday compared to professionals in the SU. The AUCwork index, which determines cortisol secretion during the work shift, was significantly lower in EU nurses. Professionals on the rotating shift, in addition to reporting a high level of stress and fatigue, also presented lower activation of the HPA axis upon awakening and the lowest levels of cortisol throughout the workday. Similarly, nurses on the morning shift presented the highest levels of stress and fatigue and the lowest levels of cortisol during the work shift and throughout the workday.

## DISCUSSION

Although data from the literature have demonstrated the importance of salivary cortisol as a neurobiological marker of occupational stress^(14)^, this study is the first to simultaneously investigate possible associations between shift work, stress, fatigue and cortisol secretion in nurses working in critical care units in hospitals.

Interpersonal relationships have been identified as the main stress factor in the work environment. In fact, interpersonal communication is one of the main causes of conflict among nurses and has a direct impact on decision-making during stressful situations^(31)^. In addition to the depersonalization of the nursing team, whose interdisciplinary work is replaced by autonomous and fragmented actions, stress caused by interpersonal conflicts promotes emotional exhaustion, feelings of distress and Burnout Syndrome^(32)^.

This study demonstrated that stress and fatigue at work are correlated constructs, and both negatively compromise the physical and mental health of professionals in health units. In nursing, work-related fatigue is a complex and multifactorial issue, generally associated with a feeling of extreme tiredness, lack of energy and reduced functional capacity^(33)^.

Coffee consumption is one of the strategies presented by nurses to remain alert and reduce fatigue symptoms, which justifies the significant and positive correlation between these variables. Moderate coffee intake can favor fundamental aspects of nurses’ cognitive performance such as attention, vigilance and reaction time^(34,35)^. Paradoxically, studies have suggested that in high concentrations, caffeine not only reduces brain vitality, but also deepens the feeling of fatigue^(36,37)^.

Desynchronization of the HPA axis is one of the main consequences of shift work, and changes in cortisol secretion are an important aspect for understanding the causal relationship between stress and adverse health effects^(38)^. The results of the present study corroborate these data, since nurses in the EU presented lower cortisol secretion in the morning, lower averages of AUCwork and AUCCD, and higher levels of stress and fatigue compared to professionals in the SU.

A study conducted with nurses demonstrated that professionals in EUs presented higher levels of stress and lower levels of salivary cortisol compared to nurses in other units^(39)^. Although in healthy individuals acute stressors elicit a strong response to cortisol, there is evidence that chronic hyperreactivity of the HPA axis promotes dysfunction in the secretion of this hormone^(40-42)^. The population of this study had, on average, 12 years of experience in the profession, so it is possible that chronic exposure to occupational stress is responsible for dysfunctions in the HPA axis and lower levels of salivary cortisol in professionals working in EUs.

Regarding work shift, nurses working on the night shift showed greater reactivity of the HPA axis upon awakening compared to professionals on the morning shift, although this was not statistically significant. This result corroborates another study of more than 7,000 workers in the United Kingdom^(43)^. Hypersecretion of this hormone after awakening has been reported in night shift workers, regardless of the number of hours worked or psychosocial stress at work.

It is known that anticipatory circumstances can interfere with the CAR in order to prepare the individual for a day with high demands^(44,45)^. In the present study, the increase in CAR in professionals working on the night shift could be the result of a state of concern about the tasks to be performed in the next few hours, when they would start their activities. The lack of association between stress, fatigue and cortisol secretion according to the work shift does not exclude the hypothesis of anticipation of CAR, since the mechanisms that promote this phenomenon are not entirely conscious and can even occur during sleep^(46)^.

To date, the role of the HPA axis in the etiology of occupational fatigue is unknown. However, several studies have demonstrated significant correlations between cortisol levels in the morning and depression, chronic fatigue and occupational stress^(4)^. These data are consistent with the results of the present study, since the AUCg indices of nurses were significantly and positively correlated with fatigue.

The importance of cortisol in energy production, metabolism, and mood, and its relationship with fatigue in clinical populations^(47)^ demonstrate the need for longitudinal studies focused on detecting this biomarker during the morning period. The cortisol response upon awakening is suitable for detecting subtle dysregulations in the HPA axis, as it is stable and little influenced by external factors^(48)^. Continuous monitoring of the effects of stress and fatigue on cortisol secretion is essential for correctly determining the harmful effects of these constructs on nurses’ health.

### Study limitations

This study has limitations that should be considered when interpreting the results. Firstly, data came from self-administered instruments, which are subject to self-report bias. It is also a predominantly female sample, which limits the possibilities of generalizing the results. However, data were collected in a homogeneous sample, collected at the same time of year and at the same times of a typical workday. These facts reduced the influence of confounding factors and increased the reliability of the results.

### Contributions to the field of Nursing

This study presents an interesting contribution to the understanding of how occupational stress and fatigue interfere with salivary cortisol secretion and, consequently, with the health of nurses in critical areas. The results presented not only corroborate the usefulness of salivary cortisol as a biomarker of occupational stress, but also point to the importance of cortisol secretion in the morning as a predictor of fatigue in nurses in critical areas.

## CONCLUSIONS

The nurses in this study presented high levels of stress and fatigue, and interpersonal relationships were the main source of stress. Although no significant associations were observed between salivary cortisol levels and occupational stressors, nurses in EUs presented higher levels of stress and fatigue and lower cortisol secretion during the workday, which suggests a dysfunction in the activity of the HPA axis. Nurses who reported fatigue had higher cortisol secretion in the morning, demonstrating the importance of this index in psychosocial research. The results indicate the need for longitudinal studies with a greater number of saliva samples collected at different times of the day and on rest days for better understanding the relationship between salivary cortisol levels, occupational stress and fatigue in nurses working in critical areas.
