# Supplementary material for: Stress and fatigue in nurses in critical care units and their association with salivary cortisol
Source: Rev Bras Enferm. 2025 Jun 20;78(2):e20240282. doi: 10.1590/0034-7167-2024-0282 (PMC12185141; doi:10.1590/0034-7167-2024-0282)
Supplement: Translation

# Translation: Estresse e fadiga em enfermeiros de unidades críticas e sua associação com o cortisol salivar

**DOI:** 10.1590/0034-7167-2024-0282pt

**Authors:** Dnieber Chagas de Assis, Deisy Vivian de Resende, Robinson Sabino-Silva, Maria Helena Palucci Marziale

**Affiliations:** I Universidade Federal de Uberlândia. Uberlândia, Minas Gerais, Brasil; II Universidade de São Paulo. Ribeirão Preto, São Paulo, Brasil

**Keywords:** Hidrocortisona, Saúde Ocupacional, Estresse Fisiológico, Fadiga, Enfermeiras e Enfermeiros.

## Abstract

**Objetivos:**

avaliar a associação entre estresse, fadiga e níveis de cortisol salivar em enfermeiros de unidades de emergência (UE) e bloco cirúrgico (BC).

**Métodos:**

estudo transversal conduzido em dois hospitais universitários. Participaram 66 enfermeiros, nos quais foram aplicados o Inventário de Estresse em Enfermeiros e a Escala de Avaliação da Fadiga e obtidas amostras de saliva para determinação da concentração do cortisol.

**Resultados:**

alto nível de estresse e fadiga foram observados em 50,8% e 46% dos profissionais, respectivamente. Enfermeiros das UE apresentaram maiores níveis de estresse e fadiga e menor secreção de cortisol salivar durante o turno de trabalho em relação aos enfermeiros do BC, sem significância estatística. A secreção de cortisol no período matutino mostrou-se correlacionada com a fadiga (ρ=0,25 e p=0,04).

**Conclusões:**

os resultados sugerem uma desregulação do eixo hipotálamo-hipófise-adrenal em enfermeiros das UE e uma correlação significativa entre fadiga e secreção de cortisol no período da manhã.

## INTRODUÇÃO

O cortisol é o principal hormônio glicocorticoide produzido pelas glândulas adrenais após ativação do eixo hipotálamo-hipófise-adrenal (HHA). Sua secreção segue uma profunda ritmicidade circadiana, com alto nível em torno de trinta minutos após acordar e gradual diminuição ao longo do dia^(1)^. Este hormônio apresenta um papel central em diversos processos biológicos, incluindo o metabolismo energético, manutenção da pressão sanguínea, imunomodulação, regulação das funções cognitivas e de memória e da resposta ao estresse. Evidências clínicas e experimentais sugerem que a concentração do cortisol pode estar relacionada com a exposição ao estresse, à fadiga e ao desenvolvimento de doenças metabólicas, e sua dosagem em amostras de saliva tem sido amplamente utilizada em pesquisas de estresse ocupacional^(2-4)^.

Profissionais de enfermagem, em virtude de longas jornadas de trabalho em diferentes turnos, estão especialmente suscetíveis ao estresse e fadiga crônicos, cujas consequências incluem adoecimento físico e mental, diminuição da capacidade para o trabalho, aumento dos acidentes ocupacionais e absenteísmo^(5,6)^. A fadiga crônica, sonolência e insônia ocasionadas pela privação/interrupção do sono são sintomas altamente prevalentes nestes profissionais, têm relação direta com o turno de trabalho e ocorrem com maior frequência em trabalhadores do período noturno^(7,8)^.

Em virtude de suas características laborais, alguns setores hospitalares apresentam maior potencial de risco ao estresse. Estudos têm demonstrado que enfermeiros de unidades de emergência (EU) e bloco cirúrgico (BC) estão expostos a estressores adicionais como ruídos, luz, radiação, violência, realização de procedimentos de ressuscitação cardiopulmonar e morte. Estímulos como necessidade de ação rápida e imediata em procedimentos de alta complexidade, superlotação e imprevisibilidade tornam enfermeiros de unidades críticas mais suscetíveis à Síndrome de Burnout, estresse psicossomático e fadiga do que aqueles de outros setores hospitalares^(3,9,10)^.

Apesar do consenso na literatura de que estressores ocupacionais promovem um desalinhamento dos ritmos circadianos e alterações endócrinas, a associação destes com a secreção salivar de cortisol e o possível impacto na saúde ainda não foram totalmente elucidados^(11-13)^, especialmente em enfermeiros de unidades hospitalares. A grande variabilidade dos desenhos metodológicos pode ser uma explicação plausível para a inconsistência dos resultados. A proliferação de índices de cortisol, muitas vezes redundantes, e o uso irracional destes construtos têm impedido a comparação entre os estudos^(14,15)^. Adicionalmente, o eixo HHA apresenta ampla funcionalidade e complexidade biológica, podendo regular situações do tipo luta e fuga, e promover adaptações comportamentais a longo prazo^(16)^.

Neste contexto, o presente estudo foi desenvolvido com o intuito de ampliar o conhecimento científico acerca das possíveis associações entre estresse ocupacional, fadiga e secreção de cortisol salivar em enfermeiros de áreas críticas. Espera-se que os resultados obtidos forneçam estratégias para a detecção de estresse e fadiga em enfermeiros, contribuindo para o desenvolvimento de medidas que visem a melhoria da saúde física e mental destes profissionais.

## OBJETIVOS

Avaliar a associação entre estresse ocupacional, fadiga e níveis de cortisol salivar em enfermeiros de UE e BC.

## MÉTODOS

### Aspectos éticos

Com base na Resolução 466 de 2012^(17)^, este estudo foi aprovado pelos Comitês de Ética em Pesquisa da Escola de Enfermagem de Ribeirão Preto da Universidade de São Paulo, do Hospital das Clínicas da Faculdade de Medicina de Ribeirão Preto da Universidade de São Paulo e da Universidade Federal de Uberlândia. O Consentimento Livre e Esclarecido por escrito foi obtido de todos os indivíduos envolvidos no estudo.

### Desenho, período e local de estudo

Trata-se de um estudo transversal com abordagem quantitativa realizado nas UE e BC de dois hospitais de ensino públicos, sendo um no estado de São Paulo (HSP) e outro no de Minas Gerais, Brasil (HMG), entre os meses de janeiro a março de 2017. As recomendações do instrumento Strengthening the Reporting of Observational Studies in Epidemiology (STROBE)^(18)^ nortearam o desenho do estudo.

### População; critérios de inclusão e exclusão

A população deste estudo constituiu-se de 191 enfermeiros e o método de amostragem utilizado foi o não probabilístico por conveniência. Foram incluídos os profissionais de ambos os sexos e que trabalhavam nos turnos matutino, vespertino e noturno, em esquemas de turno fixo ou alternante. Foi considerado turno alternante aquele que ocorria na forma de rodízio de turnos, ou seja, os enfermeiros cumpriam seu horário de trabalho tanto em turnos matutinos quanto vespertinos ou noturnos. Foram excluídos os enfermeiros que faziam uso de insulina, antiarrítmicos, medicamentos psiquiátricos ou para perda de peso, ou outros que pudessem interferir no ciclo vigília/sono ou nos níveis de cortisol, indivíduos com diagnóstico de doenças periodontais, cardiovasculares, hepáticas, psiquiátricas e distúrbios de tireoide^(19-21)^. Após a aplicação dos critérios de inclusão e exclusão, 66 enfermeiros participaram do estudo.

A primeira etapa da coleta de dados consistiu na leitura e aplicação de um instrumento composto de perguntas fechadas referentes a características sociodemográficas, atividade laboral, condições de saúde, uso de medicamentos, hábitos de vida e ciclo menstrual. Esse instrumento foi criado e validado pelos autores através do uso da técnica Delphi^(22)^, seguindo a recomendação de Spínola^(23)^ de um mínimo de 70% de concordância entre os avaliadores.

### Protocolo do estudo

A segunda etapa da coleta de dados consistiu na obtenção das amostras de saliva e na aplicação dos questionários por um membro da equipe de pesquisa. Os instrumentos foram aplicados em sala reservada, durante o expediente de trabalho de cada participante, nos turnos da manhã, tarde ou noite.

Foram aplicados o Inventário de Estresse em Enfermeiros (IEE), desenvolvido e validado para enfermeiros brasileiros por Stacciarini e Tróccoli^(24)^, para avaliação do estresse e a Escala de Avaliação da Fadiga (EAF), criada por Michielsen, de Vries, van Heck, van de Vijver, e Sijtsma^(25)^ no contexto holandês, validada para o português por Gouveia et al.^(26)^. As análises das pontuações do IEE e da EAF foram realizadas a partir da divisão do escore geral pela mediana e dicotomizaram-se os níveis de estresse em alto (maior que a mediana) e baixo (menor ou igual à mediana) e a fadiga em “presença” ou “ausência”, também com base na mediana dos dados. O IEE e a EAF apresentaram um alfa de Cronbach de 0,75 e 0,70, respectivamente.

Amostras de saliva foram coletadas em tubos Salivette^®^ (*Salimetrics, State College*, PA, EUA), de acordo com as seguintes instruções: não ingerir bebidas alcoólicas e não realizar procedimentos dentários 24h antes da coleta; não escovar os dentes ou utilizar fio dental três horas antes da coleta; não ingerir alimentos ou bebidas (exceto água) ou fumar 30 minutos antes da coleta. Imediatamente antes da coleta, checar se não há lesões orais com sangramento ativo ou potencial e higienizar a boca com água por meio de bochechos leves. Abrir o Salivette^®^, remover o *swab*, mantê-lo na boca durante dois minutos e retorná-lo à posição inicial no Salivette^®^, fechando-o firmemente. Os participantes foram orientados a manter as amostras de saliva em refrigerador e, no momento da entrega, transportá-las em isopor com gelo seco, fornecidos pela equipe da pesquisa.

Foram obtidas quatro amostras em um único dia de trabalho: ao acordar (amostra 1), 30 a 45 minutos após acordar (amostra 2), no início do turno de trabalho (amostra 3) e ao término do turno de trabalho (amostra 4). As amostras 1 e 2 foram coletadas pelo próprio participante, enquanto as demais foram coletadas por um membro da equipe da pesquisa. Adotou-se o critério estabelecido por Weitzman et al.^(27)^ de uma Resposta do Cortisol ao Acordar (CAR) mínima de 2,5 nmol/L. As amostras de saliva foram centrifugadas a 1.000×g por dois minutos a 4°C para obtenção do sobrenadante, aliquotadas e mantidas a -70ºC a até o momento da análise.

A determinação quantitativa do cortisol salivar foi realizada pelo método de imunoensaio (ELISA) do tipo competitivo, utilizando-se o kit comercial Cortisol Saliva ELISA (*Diagnostic Biochem Canada Inc*, Ontario, Canada) de acordo com as instruções do fabricante. A validação das corridas analíticas foi realizada por meio da análise da média e do desvio-padrão de cada um dos controles (alto e baixo) fornecidos no kit. Os valores de absorbância das amostras foram comparados com a curva-padrão traçada mediante análise das absorbâncias dos calibradores, e transformados em nmol/L.

A análise da atividade secretória do cortisol foi realizada de acordo com índices previamente descritos na literatura^(28,29)^. O índice AUCg representa o cálculo da área sob a curva em relação ao zero ou base, e o índice AUCi representa a área sob a curva em relação ao aumento. A secreção de cortisol durante o turno de trabalho (AUCtrab) foi calculada com base na área total sob a curva correspondente às concentrações de cortisol salivar no início e ao término de cada turno de trabalho. A secreção total de cortisol ao longo do dia de trabalho (AUCCD) foi calculada com base na área total sob a curva correspondente a todas as concentrações de cortisol ao longo do dia. Para o cálculo das áreas, o tempo foi computado em horas decimais.

### Análise dos resultados e estatística

Os dados coletados foram armazenados em um banco de dados construído no programa *Microsoft Office Excel* versão 2010. Posteriormente, foram inseridos no programa *Statical Package for the Social Sciences* (SPSS) versão 24, para as análises descritiva e inferencial. As variáveis numéricas foram categorizadas para possibilitar as análises estatísticas. As médias dos níveis de cortisol foram comparadas por meio dos testes de Mann-Whitney, para variáveis dicotômicas e Kruskall-Wallis para variáveis categóricas com mais de dois níveis, uma vez que os dados referentes ao cortisol não apresentaram distribuição normal pelo teste de Shapiro-Wilk. Para as análises de correlação entre as variáveis, utilizou-se o Coeficiente de Correlação de Spearman. As possíveis associações foram verificadas por meio dos testes Qui-quadrado e Exato de Fisher. O nível de significância adotado foi de *p* ≤ 0,05.

## RESULTADOS

Um total de 66 enfermeiros foram incluídos no estudo, sendo 51,5% (34/66) do HSP e 48,5% (32/66) do HMG. A maioria dos profissionais era do sexo feminino (75,7%; n=50), casados (60,6%; n=40) e com idade média de 38,2 ±7 anos. Com relação ao setor de trabalho, 71,2% (n=47) dos enfermeiros atuavam em UE e 28,7% no BC, com uma média de 12,7±7,4 anos de experiência na profissão e 6,5 ± 5,7 anos no setor de trabalho. Quanto ao turno de trabalho, 39,3% (n=26) dos enfermeiros estavam alocados no turno da manhã, 25,7% (n=17) no turno da tarde e 35% (n=23) no turno noturno. A maior parte dos profissionais trabalhavam em esquema de turnos alternantes (66,6%; n=44). Parte dos enfermeiros 25,7% (n=17) cumpriam carga horária semanal de trabalho superior a 44 horas.

Com relação aos hábitos de saúde, 93,9% (62/66) dos enfermeiros eram não fumantes e 56% (37/66) relataram praticar alguma atividade física. O café foi a bebida mais consumida entre os participantes (81,8%), sendo que destes, 66,6% (36/54) consumiam de uma a quatro xícaras por dia.

Dos 66 enfermeiros que coletaram as amostras de saliva, dois (3%) exibiram uma CAR menor do que 2,5nmol/L e foram considerados não aderentes ao protocolo de coleta. Um participante solicitou sua saída da pesquisa e suas amostras também foram excluídas das análises. Desta forma, a caracterização do estresse e fadiga foi realizada em 95,4% (n=63) dos participantes, sendo encontrada uma correlação significativa e positiva entre estas variáveis (ρ=0,36; p=0,03). Cerca de metade dos profissionais (50,8%) apresentaram alto índice de estresse, sendo que as relações interpessoais apresentaram maior média na somatória da pontuação (47,8±10,52). A fadiga, avaliada pelo EAF, foi observada em 46% dos enfermeiros.

Um alto índice de estresse e fadiga foram frequentemente observados em enfermeiros das UE, em profissionais de turnos alternantes e matutino, sem significância estatística (Tabela 1). Em relação às variáveis relacionadas à saúde, o consumo de café foi o único que se associou significativamente com a fadiga em enfermeiros (*p*=0,023).

**Tabela 1:** Associação entre as variáveis estresse e fadiga em relação às características ocupacionais e hábitos relacionados à saúde dos enfermeiros, Ribeirão Preto, São Paulo, Brasil, 2023 (N=63)

	Estresse			Fadiga		
	**Alto**	**Baixo**	***p***	**Presença**	**Ausência**	***p***
Local de Trabalho						
Unidade de Emergência	23	22	0,926^1^	20	25	0,783^1^
Bloco Cirúrgico	9	9		9	9	
Esquema de Trabalho						
Fixo	10	11	0,859^1^	7	14	0,187^1^
Alternante	21	21		22	20	
Turno de Trabalho						
Manhã	11	14	0,743^2^	11	14	0,930^2^
Tarde	9	7		8	8	
Noite	11	11		10	12	
Consumo de café						
Sim	24	27	0,337^1^	27	24	0,023^1^
Não	8	4		2	10	

1 Teste Qui-quadrado,

2 Teste Exato de Fisher.

Os participantes apresentaram maiores níveis de cortisol salivar no período da manhã e declínio gradual ao longo do dia^(30)^. As concentrações médias do cortisol ao acordar, 30 a 45 minutos após acordar, no início e ao término do turno de trabalho foram, respectivamente, 122,34 ± 28,19 nmol/L;140,7 ± 27 nmol/L; 110,98 ± 35,04 nmol/L e 98,03 ± 29,77 nmol/L. O aumento médio do cortisol ao acordar foi de 15% (Figura 1).

**Figura 1:** Concentração média de cortisol salivar ao longo do dia de trabalho, Ribeirão Preto, São Paulo, Brasil, 2023

Não foram evidenciadas associações estatisticamente significativas entre índices de cortisol salivar e variáveis sociodemográficas. Entretanto, indivíduos do sexo masculino apresentaram maior reatividade do eixo HHA ao acordar (AUCi) e maiores secreções de cortisol durante o turno de trabalho (AUCtrab) e ao longo do dia de trabalho (AUCCD) em comparação aos indivíduos do sexo feminino (Tabela 2). Similarmente, não foram observadas associações estatisticamente significativas entre estresse, fadiga e índices de cortisol. Entretanto, o índice AUCg, que determina a secreção total de cortisol no período da manhã, correlacionou-se significativa e positivamente com a fadiga (ρ=0,25 e *p*=0,04).

**Tabela 2:** Associação entre os índices de cortisol salivar, características sociodemográficas e ocupacionais dos enfermeiros, Ribeirão Preto, São Paulo, Brasil (N=63)

	AUCi	*p*	AUCg	*p*	AUCTrab	*p*	AUCCD	*p*
Sexo								
Feminino	4,79 (±3,70)	0,170^1^	75,36 (±24,73)	0,583^1^	812,63 (±424,76)	0,232^1^	2046,17 (±1242,48)	0,821^1^
Masculino	6,14 (±4,23)		65,80 (±22,90)		952,66 (±426,30)		2364,02 (±1726,74)	
Estresse								
Baixo	5,54 (±4,08)	0,329^1^	72,53 (±15,00)	0,705^1^	832,76 (±385,73)	0,967^1^	2064,45 (±1324,23)	0,773^1^
Alto	4,7 (±3,63)		73,62 (±31,07)		858,78 (±467,45)		2177,46 ( ±1421,57)	
Fadiga								
Presença	5,15 (±4,08)	0,983^1^	75,11 (±17,65)	0,247^1^	912,50 (±487,37)	0,432^1^	1998,76 (±1456,25)	0,424^1^
Ausência	5,09 (±3,88)		71,36 (±29,01)		789,24 (363,48)		2226,84 (±1294,07)	
Esquema de Trabalho								
Fixo	5,92 (±4,43)	0,351^1^	68,91 (±21,68)	0,281^1^	851,71 (±421,92)	0,942^1^	2199,18 (±1457,68)	0,816^1^
Alternante	4,71 (±3,50)		75,16 (±25,54)		843,10 (±432,99)		2083,18 (1332,24)	
Turno de Trabalho								
Manhã	4,59 (±3,01)	0,763^2^	71,34 (±14,58)	0,522^2^	833,47 (±436,57)	0,981^2^	1896,25 (±1419,61)	0,125^2^
Tarde	5,04 (±4,24)		78,29 (±24,40)		868,61 (±461,99)		2356,32 (±890,76)	
Noite	5,76 (±4,43)		71,27 (32,43)		843,71 (±405,91)		2207,68 (±1586,72)	

1 Teste Mann-Whitney,

2 Teste Kruskall-Wallis.

Enfermeiros das UE apresentaram menores índices de cortisol salivar durante todo o dia de trabalho, quando comparados aos profissionais do BC. O índice AUCtrab, que determina a secreção de cortisol durante o turno de trabalho, foi significativamente menor em enfermeiros de UE. Profissionais do turno alternante, além de relatarem um alto índice de estresse e fadiga, também apresentaram uma menor ativação do eixo HHA ao acordar e os menores índices de cortisol durante todo o dia de trabalho. Similarmente, enfermeiros do turno matutino apresentaram os mais altos índices de estresse e presença de fadiga e menores índices de cortisol durante o turno de trabalho e durante todo o dia de trabalho.

## DISCUSSÃO

Embora dados da literatura tenham demonstrado a importância do cortisol salivar como marcador neurobiológico do estresse ocupacional^(14)^, o presente estudo é o primeiro a investigar concomitantemente possíveis associações entre o trabalho em turnos, estresse, fadiga e secreção de cortisol em enfermeiros de unidades críticas hospitalares.

As relações interpessoais foram apontadas como o principal fator estressante no ambiente de trabalho. De fato, a comunicação interpessoal é uma das principais causas de conflito entre enfermeiros e tem impacto direto na tomada de decisões durante situações estressantes^(31)^. O estresse por conflitos interpessoais promove, além da descaracterização da equipe de enfermagem, cujo trabalho interdisciplinar é substituído por ações autônomas e fragmentadas, exaustão emocional, sentimentos de angústia e Síndrome de Burnout^(32)^.

O presente estudo demonstrou que estresse e fadiga no trabalho são construtos correlacionados, de modo que ambos comprometem negativamente a saúde física e mental dos profissionais de unidades de saúde. Em enfermagem, a fadiga relacionada ao trabalho é uma questão complexa e multifatorial, geralmente associada a uma sensação de extremo cansaço, falta de energia e reduzida capacidade funcional^(33)^.

Acredita-se que o consumo de café seja uma das estratégias apresentadas pelos enfermeiros para manterem-se alertas e diminuírem os sintomas da fadiga, o que justifica a correlação significativa e positiva entre estas variáveis. A ingestão moderada de café pode favorecer aspectos fundamentais da performance cognitiva dos enfermeiros como atenção, vigilância e tempo de reação^(34,35)^. Paradoxalmente, estudos têm sugerido que, em altas concentrações, a cafeína não apenas reduz a vitalidade cerebral, como também aprofunda a sensação de fadiga^(36,37)^.

A dessincronização do eixo HHA é uma das principais consequências do trabalho em turnos, e alterações na secreção de cortisol constituem aspecto importante para o entendimento da relação causal entre estresse e efeitos adversos na saúde^(38)^. Os resultados do presente estudo corroboram estes dados, uma vez que enfermeiros das UE apresentaram menor secreção de cortisol no período da manhã, menores médias de AUCtrab e AUCCD, e maiores índices de estresse e fadiga em comparação aos profissionais do BC.

Um estudo realizado com enfermeiros demonstrou que profissionais de UE apresentavam maiores níveis de estresse e menores níveis de cortisol salivar comparativamente aos enfermeiros de outras unidades^(39)^. Embora em indivíduos saudáveis estressores agudos elicitem forte resposta ao cortisol, há evidências de que a hiper-reatividade crônica do eixo HHA promova disfunção na secreção deste hormônio^(40-42)^. Como a população deste estudo apresentava, em média, 12 anos de profissão, é possível que a exposição crônica ao estresse ocupacional seja responsável por disfunções no eixo HHA e menores níveis de cortisol salivar em profissionais das UE.

Em relação ao turno de trabalho, enfermeiros do turno noturno apresentaram maior reatividade do eixo HHA ao acordar em comparação aos profissionais do turno matutino, no entanto, sem significância estatística. Este resultado corrobora outro estudo com mais de 7.000 trabalhadores do Reino Unido^(43)^. Foi relatada a hipersecreção deste hormônio após acordar em trabalhadores do turno noturno, independentemente do número de horas trabalhadas ou de estresse psicossocial no trabalho.

Sabe-se que circunstâncias antecipatórias podem interferir na CAR com o objetivo de preparar o indivíduo para um dia com altas demandas^(44,45)^. No presente estudo, o aumento da CAR em profissionais do turno noturno poderia ser resultado de um estado de preocupação pelas tarefas a serem realizadas nas próximas horas, quando os mesmos iniciariam atividades. A ausência de associação entre estresse, fadiga e secreção de cortisol de acordo com o turno de trabalho não exclui a hipótese de antecipação da CAR, pois os mecanismos que promovem tal fenômeno não são de natureza totalmente consciente, podendo ocorrer, inclusive, durante o sono^(46)^.

Até o momento, não se conhece o papel do eixo HHA na etiologia da fadiga ocupacional. Entretanto, diversos estudos têm demonstrado correlações significativas entre os níveis de cortisol no período da manhã e depressão, fadiga crônica e estresse ocupacional^(4)^. Tais dados são consistentes com os resultados do presente estudo, uma vez que os índices AUCg dos enfermeiros mostraram-se significativa e positivamente correlacionados com a fadiga.

A importância do cortisol na produção de energia, metabolismo e humor e de sua relação com a fadiga em populações clínicas^(47)^ demonstra a necessidade de estudos longitudinais focados na detecção deste biomarcador durante o período da manhã. A resposta do cortisol ao despertar é adequada para detecção de desregulações sutis no eixo HHA, pois é estável e sofre pouca influência de fatores externos^(48)^. O monitoramento contínuo dos efeitos do estresse e fadiga na secreção de cortisol é imprescindível para a correta determinação dos efeitos nocivos destes construtos sobre a saúde dos enfermeiros.

### Limitações do estudo

O presente estudo apresenta limitações que devem ser consideradas ao interpretar os resultados. Primeiramente, os dados obtidos são provenientes de instrumentos autoaplicáveis estando, por sua vez, sujeito a viés de autorrelato. Trata-se também de um universo amostral majoritariamente feminino, o que limita as possibilidades de generalização dos resultados. No entanto, os dados foram realizados em uma amostra homogênea, coletada na mesma época do ano e nos mesmos horários de um dia típico de trabalho. Tais fatos reduziram a influência de fatores de confundimento e aumentaram a confiabilidade dos resultados.

### Contribuições para a área da Enfermagem

O presente estudo apresenta interessante contribuição para o entendimento de como o estresse ocupacional e a fadiga interferem na secreção de cortisol salivar e, consequentemente, na saúde de enfermeiros de áreas críticas. Os resultados apresentados não apenas corroboram a utilidade do cortisol salivar como biomarcador de estresse ocupacional, como também apontam a importância da secreção de cortisol no período da manhã como preditor de fadiga em enfermeiros de áreas críticas.

## CONCLUSÕES

Os enfermeiros do presente estudo apresentaram altos índices de estresse e fadiga, sendo as relações interpessoais a principal fonte de estresse. Embora não tenham sido observadas associações significativas entre níveis de cortisol salivar e estressores ocupacionais, enfermeiros das UE apresentaram maiores índices de estresse e fadiga e menor secreção de cortisol durante o dia de trabalho, o que sugere uma disfunção na atividade do eixo HHA. Enfermeiros que relataram fadiga apresentaram uma maior atividade secretória de cortisol no período da manhã, demonstrando a importância deste índice em pesquisas psicossociais. Os resultados apontam para a necessidade de estudos longitudinais, com um maior número de coletas de saliva em diferentes momentos do dia e em dias de descanso, para um melhor entendimento da relação entre níveis de cortisol salivar, estresse ocupacional e fadiga em enfermeiros de áreas críticas.
